# Applying interprofessional Team-Based Learning in patient safety: a pilot evaluation study

**DOI:** 10.1186/s12909-018-1164-8

**Published:** 2018-03-27

**Authors:** Lukas Lochner, Sandra Girardi, Alessandra Pavcovich, Horand Meier, Franco Mantovan, Dietmar Ausserhofer

**Affiliations:** 1 Claudiana – College of Healthcare Professions, Via Lorenz Böhler 13, 39100 Bolzano, Bozen, Italy; 2 South Tyrolean Health Trust, Bolzano, Bozen, Italy; 3 Ministry of Health (Department 23 – Healthcare), Clinical Governance, Bolzano, Bozen, Italy; 4 Department of Public Health, Institute of Nursing Science, University of Basel, Basel, Switzerland

**Keywords:** Team-based learning, Interprofessional education, Patient safety, Learning from errors, Pre-qualifying non-medical healthcare students

## Abstract

**Background:**

Interprofessional education (IPE) interventions are not always successful in achieving learning outcomes. Team-Based Learning (TBL) would appear to be a suitable pedagogical method for IPE, as it focuses on team performance; however, little is known about interprofessional TBL as an instructional framework for patient safety. In this pilot-study, we aimed to (1) describe participants’ reactions to TBL, (2) observe their achievement with respect to interprofessional education learning objectives, and (3) document their attitudinal shifts with regard to patient safety behaviours.

**Methods:**

We developed and implemented a three-day course for pre-qualifying, non-medical healthcare students to give instruction on non-technical skills related to ‘learning from errors’. The course consisted of three sequential modules: ‘Recognizing Errors’, ‘Analysing Errors’, and ‘Reporting Errors’. The evaluation took place within a quasi-experimental pre-test-post-test study design. Participants completed self-assessments through valid and reliable instruments such as the Mennenga’s TBL Student Assessment Instrument and the University of the West of England’s Interprofessional Questionnaire. The mean scores of the individual readiness assurance tests were compared with the scores of the group readiness assurance test in order to explore if students learned from each other during group discussions. Data was analysed using descriptive (i.e. mean, standard deviation), parametric (i.e. paired t-test), and non-parametric (i.e. Wilcoxon signed-rank test) methods.

**Results:**

Thirty-nine students from five different bachelor’s programs attended the course. The participants positively rated TBL as an instructional approach. All teams outperformed the mean score of their individual members during the readiness assurance process. We observed significant improvements in ‘communication and teamwork’ and ‘interprofessional learning’ but not in ‘interprofessional interaction’ and ‘interprofessional relationships.’ Findings on safety attitudes and behaviours were mixed.

**Conclusion:**

TBL was well received by the students. Our first findings indicate that interprofessional TBL seems to be a promising pedagogical method to achieve patient safety learning objectives. It is crucial to develop relevant clinical cases that involve all professions. Further research with larger sample sizes (e.g. including medical students) and more rigorous study designs (e.g. pre-test post-test with a control group) is needed to confirm our preliminary findings.

**Electronic supplementary material:**

The online version of this article (10.1186/s12909-018-1164-8) contains supplementary material, which is available to authorized users.

## Background

International studies in acute hospitals reveal that, on average, around 10% of patients experience one or more adverse events (e.g. healthcare-associated infections, patient falls), and half of these events are preventable [1]. Healthcare professionals work in a complex and high-risk system and are confronted with making and observing errors: unsafe acts in the process of care that can lead to critical incidents, i.e. near misses or adverse events [2]. In order to prevent errors and consequent negative health outcomes, both technical and non-technical skills are fundamental for healthcare professionals. Technical skills refer to professional knowledge and abilities (e.g. basic life support and cardiopulmonary resuscitation). Non-technical skills refer to communication, teamwork, and leadership [3–6]. Recent studies have emphasized that non-technical skills are important core competencies for healthcare professionals to help them prevent errors and encourage learning from errors [7, 8].

Interprofessional education (IPE) is gaining increasing worldwide recognition as a way to enhance healthcare professionals’ non-technical skills [9]. In IPE two or more health professionals learn with, from, and about each other to improve collaboration and the quality of care [10]. There is growing evidence that IPE can have a positive impact on working collaboratively in clinic, thus improving patient care and reducing error rates [11–16]. However, IPE is also criticized as sometimes failing to generate positive changes in attitude with respect to interprofessional communication and teamwork [17, 18]. If the educational design of interventions is poor, then mandating students from different programs to spend time together can be counter-productive [19]. In order to avoid counter-productive changes, students need to be engaged in meaningful learning activities.

Experts recommend introducing IPE into undergraduate programs as a way to reduce students’ prejudices towards other healthcare professions and prepare them for the complexity of professional socialization and teamwork, rather than simply assuming that these skills will be acquired later on in their clinical positions [13, 20, 21]. In order to enable them to understand the contribution that effective interprofessional collaboration makes to the delivery of safe and high-quality care, their attention needs to be drawn not only to the content but also to the process of learning. Students should interact with each other in a way that fosters shared decision-making and listening to other team members [13, 15].

A pedagogical method that seems suitable for IPE in the field of patient safety is Team-Based Learning (TBL), an instructional approach that focuses on the application and integration of information and that allows for assessments of both individual and team performance [22, 23]. TBL combines multiple small groups into a single classroom, where students master content through three steps: (1) individual out-of-class preparation (e.g. textbooks, lecture videos); (2) in-class multiple choice test first administered to individual learners (Individual Readiness Assurance Test, IRAT) and then to assigned teams (Group Readiness Assurance Test, GRAT). These tests hold learners accountable for Step 1 and foster peer-to-peer teaching in areas of deficiency; (3) in-class application of content through team problem-solving activities in which course content is presented in real-world scenarios. Steps 2 and 3 are facilitated by a content expert and typically involve intra- and inter-group discussions of the course material. All teams in the class work on the same problem at the same time and share their solutions simultaneously. This provides an excellent vehicle for student teams to work collaboratively. Responsibility is divided, which helps with group learning during in-class discussions. Since TBL also draws the attention of participants to the process of learning, it has been correlated with encouraging better communication and teamwork skills [24, 25].

Understanding IPE in the context of patient safety has been the focus of recent research [14, 26–28], and there is a contemporary body of literature on educational interventions that relates to training in non-technical skills [6, 29]. Recent studies provide evidence that undergraduate IPE experiences can enhance readiness for interprofessional learning, attitudes to teamwork and engagement in patient safety issues [30, 31]. However, to our knowledge, no previous study has applied interprofessional TBL as an instructional framework for patient safety.

### Aims

In this pilot-study we aimed to investigate the effects of interprofessional TBL for pre-qualifying, non-medical healthcare students with regard to (1) the students’ reaction to the didactic approach (i.e. TBL methodology); (2) induced changes in student perception of interprofessional education (i.e. communication and teamwork, interprofessional learning, interprofessional interactions, and interprofessional relationships); and (3) changes in students’ attitudes to patient safety. The following research questions guided our study:

1. How do students react to interprofessional TBL?
2. Do students’ perceptions of interprofessional education change?
3. Do students’ attitudes towards patient safety issues change?

## Methods

### Design

We developed an “interProfessional Education in Patient Safety (iPEPS)” course on ‘learning from errors’, an educational pilot project for pre-qualifying, non-medical healthcare students, in which we adapted and applied the TBL methodology. To evaluate the effect of the course on students, we applied a quasi-experimental study designed as a pre-test-post-test without a control group.

### Educational setting

The Claudiana – College of Healthcare Professions in Bolzano/Bozen, northern Italy, offers three-year bachelor’s programs in 12 non-medical health professions (nursing, obstetrics, physiotherapy, dietetics and nutrition, occupational therapy, speech therapy, radiology techniques, laboratory techniques, environmental technology, orthoptics, dental hygiene and podiatry). Currently, no medical students are being trained at our institution. The programs are affiliated with four Italian universities (University of Verona, University of Ferrara, Catholic University of Rome, Sapienza University of Rome). The predominant teaching method is didactic lecturing to groups of 20 to 120 students. Approximately 650 students are enrolled in the various programs. The curricula of the programs are strictly segregated, and neither IPE nor TBL have been implemented so far.

### Development and implementation of the course

The development of the iPEPS course followed Kern’s approach for curriculum development [32]. The TBL sessions are described based on the guidelines for reporting TBL activities [33].

First of all, we assessed Claudiana’s bachelor’s healthcare curricula with course directors and found that, although elements of patient safety and risk management are taught, there was a lack of teaching related to ‘learning from errors’. Following the WHO’s Multi-professional Patient Safety Curriculum Guide [34], we developed a course to foster learning from errors. The course teaches students to (1) apply systems thinking principles to recognize care delivery problems and factors contributing to critical incidents; (2) report critical incidents via appropriate systems in a timely manner; and (iii) propose improvement strategies to avoid the reoccurrence of incidents. The specific learning objectives and content of the iPEPS course are described in more detail in Table 1.

**Table 1:** Outline of the iPEPS-course

	Module 1 (4 h)	Module 2 (4 h)	Module 3 (4 h)
1. Learning objectives (What will students be able to do?)	(1) Define the most relevant key terms, concepts and models in the field of ‘patient safety.’ (2) Explain the reasons and most important contributing factors for the occurrence of critical incidents in healthcare organizations. (3) Reflect on their personal attitude towards critical incidents and how these are managed within an interprofessional team. (4) Recognize the importance of developing a positive and open safety and error-free culture for their professional work.	(1) Describe the associations between error-contributing (system) factors and critical incidents, as well as strategies to avoid their reoccurrence. (2) Compute an error and risk analysis of a critical incident based on the Accident Causation Model (“Swiss Cheese” Model) and according to the London protocol. (3) Recognize the perspectives and roles within an interprofessional team.	(1) Decide which critical incidents need to be reported and justify the decision within an interprofessional team. (2) Correctly fill out a critical incident report form of the South Tyrolean Health Care Trust with an interprofessional team. (3) Reflect on which factors can foster or hinder the reporting of critical incidents in healthcare organizations.
2. Content (What will students learn?)	- Key terms, concepts and models (e.g. types of errors, critical incident, near miss, adverse event, safety/error culture, Accident Causation Model (“Swiss Cheese” Model). - The difference between errors, critical incidents and near miss, adverse and sentinel events. - Human factors: reasons for and contributing factors on the individual and organizational level to the occurrence of critical incidents and patient harm. - Methods and tools for recognizing and intervening in potential critical situations (speaking up).	- Accident Causation Model (“Swiss Cheese” Model). - Error and risk analysis according to the London-Protocol.	- The reporting and learning system of the South Tyrolean Health Care Trust (*CIRS*) to improve patient safety.
3. Didactic methods (TBL) (How will students learn?)	*TBL Phase 1: Pre-class study* - iPEPS pre-test - Literature (book chapters) *TBL Phase 2: Readiness Assurance* - IRAT/GRAT (multiple choice test) - Appeals/Feedback *Slide and lecture (15 min)* - ‘Recognizing errors’ (key messages) *TBL Phase 3: Application* - Group assignments based on case studies (speaking up/observing care delivery problems) - Groups make specific choices, followed by simultaneous reporting.	- Watch WHO video on Vincrestine - Literature (London Protocol) - IRAT/GRAT (multiple choice test) - Appeals/Feedback - Analysing errors (key messages) Group work (error and risk analyses of an adverse event using fishbone diagram / proposing quality improvement interventions), followed by sequential presentation in plenum.	- Critical Incident Report Form - Literature (articles) - IRAT/GRAT (multiple choice test) - Appeals/Feedback - Reporting errors (key messages) Group work (Case scenarios / decision making on which critical incident needs to be reported / filling out the critical incident report form), followed by sequential presentation in plenum.
4. Assessment (How will learning outcomes be evaluated?)	- Individual Performance (IRAT) - Team Performance (GRAT)	- Individual Performance (IRAT) - Team Performance (GRAT)	- Individual Performance (IRAT) - Team Performance (GRAT)

The course was organized into three sequential modules (‘Recognizing errors’, ‘Analysing errors’, and ‘Reporting errors’). The overall student workload was 12 h. For each module, 1 h was allocated to pre-class preparation, followed by 3 h of TBL sessions. The sessions were led by one or two content experts and facilitated by a medical educator; the role of the latter was to ensure that the content experts could focus on the subject matter, rather than on the formal TBL process. Each session began with an IRAT, which consisted of five to 14 multiple-choice items. After the answer sheets were collected, students completed the GRAT by taking the same test as a team. Following the GRAT, teams revealed their solutions simultaneously, step-by-step, holding up cards to expose their answers. Then the medical educator moderated a debriefing, allowing for immediate feedback and discussion between the teams and the expert. Before distributing the application exercises, the expert provided a 10- to 15-min summary of the core messages to ensure a thorough understanding of the modules’ concepts. We considered this an appropriate measure to guarantee adequate preparedness for the exercises, as students and teachers had no prior practical experience with TBL. A sample case is provided in Additional file 1.

In the application for Module 1 (‘Recognizing errors’), teams were asked to label the events as an error, near miss, critical incident or sentinel event, and/or select the most appropriate safety behaviour from a given list. The team solutions were reported simultaneously to the whole class, followed by immediate feedback. Each team worked on the same problems to make specific choices. However, during the application phase for Module 2 (‘Analysing errors’) and 3 (‘Reporting errors’), the group assignments were adapted to simulate targeted real world experiences. Teams received the clinical scenarios on paper, which they had to analyse by filling out a predefined scheme (fishbone diagram). In Module 3, they had to complete the form sheet of the local critical incident reporting system and results were not reported simultaneously, but presented sequentially in plenum and discussed together with the experts. As this was a pilot project that has not yet been implemented into the curriculum, no grading was performed and no peer review was integrated. However, the IRATs and GRATs results were analysed.

We invited second and third (final) year students to participate in the course. The course was limited to a maximum of 42 participants due to the size of the available classroom suitable for TBL activities. Participation was awarded on a ‘first-come, first-serve’ basis. Students were stratified according to their professional group and randomly allocated to one of the six teams, each containing six or seven members. The preparatory assignments (e.g. textbooks, videos) were available through a web-based learning system.

### Evaluation of the course

Students filled out structured questionnaires on paper 2 to 3 weeks before the course and 1 week after it. Filling out the questionnaires was part of the coursework and a prerequisite for the successful completion of the course. The students’ socio-demographic characteristics (gender, age, course, education year) were assessed in the pre-test questionnaire. The students’ preference for lecture or Team-Based Learning was assessed in the post-test questionnaire, using six items of the corresponding 16-item subscale of the Team-Based Learning Student Assessment Instrument (TBL-SAI) developed by Mennenga [35].

The following variables and measures were included in the pre-test and post-test questionnaire: ‘Students’ attitudes towards interprofessional education’ was measured with the German version of the University of the West of England’s Interprofessional Questionnaire (UWE-IP-D), developed by Pollard et al. [20]. This instrument consists of four subscales: *communication and teamwork* (nine items), *interprofessional learning* (nine items), *interprofessional interactions* (nine items), and *interprofessional relationships* (eight items). Each subscale achieved acceptable levels of internal consistency (α = 0.92–0.52). ‘Students’ patient safety attitudes’ were measured with 18 items selected from the students evaluation questionnaires of the WHO’s Patient Safety Curriculum Guide for Medical School [7]. All instruments and items used a five-point Likert scale ranging from “strongly disagree” to “strongly agree.” All instruments and items (except the German version of the UWE-IP-D) were translated from English to German by one member of the project group and checked by another. The face validity of the questionnaire was assessed within the project team.

### Data collection and analysis

Students generated a unique code (the first three letters of their mother’s surname, the first two letters of their mother’s first name, the last two numbers of their mother’s birth year) that allowed us to connect their pre-test and post-test questionnaires. Data collected from the questionnaires was entered into IBM SPSS Statistics 21.0 (IBM Corp., Armonk, NY). First, all reverse-coded items were recoded and then analysed using appropriate descriptive analyses, including absolute and relative frequencies of positive responses (scores 4 and 5), as well as means and standard deviations. Differences between the students’ perceptions of TBL and didactic lecturing, as well as pre-test and post-test scores concerning interprofessional education and patient safety attitudes, were analysed using parametric (i.e. paired t-test) or non-parametric (i.e. Wilcoxon signed rank test) analyses. The statistical significance was set at a *p*-value of less than or equal to 0.05.

## Results

### Participant demographics and response rate

A total of 39 students completed the entire course, with representation from five different programs (nursing, dietetics and nutrition, occupational therapy, radiology techniques, laboratory techniques). Table 2 shows the participant demographics. As completing the pre-questionnaire was a prerequisite for participation, and completion of the post-questionnaire was required to obtain the participation certificate, we reached a response rate of 100%.

**Table 2:** Participant demographics (*N* = 39)

Age in years: Mean (SD)	22.65 (2.8)
Gender: F:M	37:2
Professional program: *n*	Nursing: 15 Occupational therapy: 9 Laboratory techniques: 7 Dietetics and nutrition: 6 Physiotherapy: 1 Radiology techniques: 1
Year of program: *n*	First year: - Second year: 15 Third (final) year: 24

### How did students react to the introduction of TBL in an interprofessional setting?

In the post-test questionnaire students were asked to what extent they preferred the newly introduced TBL approach over the didactic lectures to which they were accustomed. Table 3 shows that for the items relating to ‘retention’ and ‘self-study’ students reported higher scores (84.6 and 64.1% gained scores of 4 and 5) for TBL, with the difference being significant (*p* < 0.05). Findings for the item ‘distraction’ indicate that fewer students reported to be distracted during TBL sessions compared to didactic lectures. However, there was no statistically significant difference.

**Table 3:** Evaluation of preference for lecture or Team-Based Learning approach (*N* = 39)

1: Traditional lecture			2: Team-Based learning			1 vs 2 Significance, Wilcoxon test
Questions	% 4 + 5 (Agree + Strongly agree)	Mean (SD)	Questions	% 4 + 5 (Agree + Strongly agree)	Mean (SD)	
Distraction						
*During traditional lectures, I often find myself thinking of non-related things.* ^a^	20.5%	2.67 (0,98)	*I talked about non-related things during Team-Based Learning activities.* ^a^	38.5%	2.95 (0.92)	*P* = 0.076 Z = −1.77
Retention						
*I remember material better when the instructor lectures about it*.	20.5%	2.67 (0.87)	*I remember material better when I used it during Team-Based Learning activities.*	84.6%	4.03 (0.67)	*P* < 0.001 Z = −4.62
Self-study						
*It is easier to study for an exam when the instructor has lectured about the material.*	7.9%	2.47 (0.89)	*I would do better on exams if we used Team-Based Learning to cover the material.*	64.1%	3.67 (0.81)	*P* < 0.001 Z = − 4.24

^a^ item reversed for analysis (a higher score means lower students’ distraction)

To explore if students learned from each other during group discussions, the mean scores of the IRAT (administered to all group members individually) were compared with the scores of the GRAT (administered during group discussion; answers were chosen based on consensus decision). All teams outperformed the mean score of their individual team members, with the average mean difference being 1.41 points (10.1%). In one group, however, the best team member did better than the group. Table 4 displays Module 1’s IRAT mean score and GRAT score. With 14 multiple-choice items, Module 1 featured the most extensive Readiness Assurance Process of the three modules.

**Table 4:** Score changes from IRAT to GRAT (*N* = 39)

Team no	IRAT (max. 14 points) Mean (SD)	GRAT (max. 14 points)	IRAT to GRAT Mean difference
1 (*n* = 6)	10.33 (1.21)	12	+ 1.67
2 (*n* = 6)	10.67 (1.03)	12	+ 1.33
3 (*n* = 7)	10.71 (1.11)	12	+ 1.29
4 (*n* = 7)	10.92 (1.98)	11	+ 0.08
5 (*n* = 7)	11.43 (1.72)	13	+ 1.57
6 (*n* = 6)	9.50 (1.22)	12	+ 2.50
TOTAL	10.59 (0.65)	12	+ 1.41

### Did students’ perceptions of interprofessional education change?

The UWE-IP-D Interprofessional Questionnaire focuses on attitudinal shifts on four subscales: *(1) communication and teamwork*, *(2) interprofessional learning*, *(3) interprofessional interaction*, and *(4) interprofessional relationships*. Table 5 indicates that significant positive changes occurred for the first two subscales (*p* < 0.05). Shifts in attitude towards interprofessional interaction and relationships were not significant.

**Table 5:** Pre- and post-results of the UWE-IP-D Interprofessional Questionnaire (*N* = 39)

Subscales (scale range)	Pre Mean (SD)	Post Mean (SD)	Pre vs post Significance, paired t-test
*Communication and Teamwork* (9–36)	21,46 (5,58)	23,59 (5,62)	*P* = 0.038 *T* = −2.16
*Interprofessional Learning* (9–45)	33,97 (6,16)	36,36 (5,68)	*P* = 0.036 *T* = − 2.17
*Interprofessional Interaction* (9–45)	25,82 (3,69)	25,77 (3,98)	*P* = 0.952 *T* = 0.06
*Interprofessional Relationships* (8–40)	29,26 (3,63)	30,87 (3,78)	*P* = 0.062 *T* = −1.92

### Did students’ attitudes towards patient safety issues change?

Table 6 shows mixed results for the pre-test post-test comparison of seven safety attitudes and behaviours. While the scores of some items (e.g. ‘filling in reporting forms will help to improve patient safety’) increased as expected from pre- to post-test, the scores of other items (e.g. ‘telling others about an error I made would be easy’) surprisingly declined. However, none of these changes were statistically significant.

**Table 6:** Selected pre- and post-results of the students evaluation questionnaires of the WHO’s Patient Safety Curriculum Guide (*N* = 39)

Questions	Pre % 4 + 5 Mean (SD)	Post % 4 + 5 Mean (SD)	Pre vs post Significance, Wilcoxon test
*If I keep learning from my mistakes, I can prevent incidents.*	89.7% 4.41 (0.85)	82.1% 4.21 (0.80)	P = 0.16 Z = − 1.40
*Acknowledging and dealing with errors will be an important part of my job.*	89.7% 4.33 (0.84)	87.2% 4.13 (0.61)	*P* = 0.21 Z = − 1.26
*Telling others about an error I made would be easy.*	43.6% 3.31 (0.98)	28.2% 3.10 (0.79)	*P* = 0.16 Z = − 1.41
*It is easier to find someone to blame rather than focus on the causes of error.*	33.3% 2.85 (1.11)	17.9% 2.64 (1.04)	*P* = 0.36 Z = − 0.91
*I am always able to ensure that patient safety is not compromised.*	35.9% 3.18 (0.89)	23.7% 2.84 (0.92)	*P* = 0.09 Z = − 1.67
*I believe that filling in reporting forms will help to improve patient safety.*	66.7% 3.72 (0.97)	84.6% 3.95 (0.60)	*P* = 0.30 Z = − 1.05
*I plan to inform my colleagues about the errors they make.*	64.1% 3.62 (0.75)	100% 3.77 (0.43)	*P* = 0.31 Z = − 1.02

## Discussion

This pilot-study aimed to evaluate the course “interProfessional Education in Patient Safety (iPEPS)”. Our findings indicate that interprofessional TBL was well received by the students as an instructional approach to fostering learning about ‘learning from errors’. All teams outperformed the mean score of their individual members. The course yielded significant improvements in students’ perceptions toward ‘communication and teamwork’ and ‘interprofessional learning’.

Students rated TBL significantly higher than didactic lecturing for ‘retention’ and ‘self-study.’ However, their attention span during group work presentations may not have been entirely satisfactory, as we did not observe significant differences for ‘distraction’. This constitutes a matter that needs improvement ([23], p.53). During Modules 2 and 3, teams worked on different case scenarios and reporting took place sequentially, not simultaneously. We experienced that building realistic case scenarios that engage all the participating healthcare professions is crucial for interprofessional TBL. This resonates with statements of other authors who found that, when planning IPE initiatives, particular focus is needed to make sure that disciplinary knowledge is necessary in a way that all students are highly motivated to contribute to the learning activity [30, 36]. Yet this is challenging in the field of patient safety as there are few critical incidents or adverse events that equally engage a high number of different healthcare professionals. Both aspects could explain students’ distraction during the reporting of group work. However, all teams outperformed the mean score of their individual team members during the Readiness Assurance Process, which indicates that group discussion was beneficial for at least the weaker group members.

We observed significant improvements in students’ perception towards *‘communication and teamwork’* and *‘interprofessional learning’*, providing first evidence that the course increased students’ positive attitude towards communicating with other professions and working in teams. IPE initiatives are not always successful in producing attitudinal changes, since mandating students to spend time together can prove counter-productive [17, 18]. Judge et al. [30] investigated students’ readiness for interprofessional learning after the exposure of 308 students from different health care programs to interprofessional learning activities (i.e., PowerPoint presentations and case-based exercises). This study revealed that interprofessional education activities require a student-centred teaching strategy rather that a presentation based intervention [30]. Our findings suggest that TBL constitutes such a methodology as it supported the achievement of important IPE objectives. TBL was not compared to another educational approach in this study, but free-text comments in the post-course questionnaire confirmed that participants greatly valued the contact and interaction with students from different professional backgrounds that were stimulated by the team assignments. However, we did not find any improvements in *‘interprofessional interaction’* and *‘interprofessional relationships’*. This might indicate that the course was too short. The interaction between the participants was limited to 3 days and most likely ended at the end of the course. Well-designed group assignments can produce positive outcomes, but it is only when students work together over an extended period of time that their groups can develop into teams in which communication becomes more open and conducive to learning ([23], p.11). Although we were able to foster learning with and from each other, we were less successful at fostering learning about each other, e.g. about the different health professions’ roles and clinical tasks. Here again, it becomes evident that the design of the clinical cases must ensure that disciplinary knowledge of all participating professions is necessary in a way that learning about each other is fostered by the requirements of the activity [19, 36].

Findings regarding students’ attitudes towards patient safety behaviours were mixed. For example, from pre- to post-test, fewer students reported that telling others about an error was easy. It seems that students became more aware of how challenging it is to deal with patient safety during daily professional routines. This new finding has not yet been reported in the literature. We need to be aware that educating students on ‘learning from errors’ might lead to expectations and attitudes towards critical incident reporting that might reduce their willingness to report errors. This would be undesirable as critical incident reporting systems are often underused in clinical practice [37]. Students’ willingness to identify, report, and analyse errors in their future clinical posts needs to be investigated in follow-up studies.

### Limitations

The findings need to be interpreted in light of several limitations. Applying a traditional pre-test post-test comparison without a control group and the small sample size affects the internal validity of the study. As we did not compare TBL to other forms of educational interventions, causal interpretation of the effects of TBL is not justified. Since students compared the iPEPS course to their normal coursework, their positive reaction to the introduction of TBL might have been affected by the topic, the teachers and/or the type of course material. Furthermore, there is a selection bias. As participation in the course was voluntary, only highly-motivated students interested in IPE and the topic of patient safety participated in the course, leading to high pre-test values.

## Conclusion

This pilot study investigated interprofessional TBL about patient safety. It revealed significant improvements in students’ perceptions towards *‘communication and teamwork’* and *‘interprofessional learning’.* Interprofessional TBL appears to be a promising pedagogical method to achieve patient safety learning objectives. Design of clinical cases to include all participating professions seems to be crucial to support the achievement of learning outcomes. Qualitative research with focus groups should further explore how interprofessional TBL fosters students’ learning and teamwork. To confirm our preliminary findings, further quantitative research is required with larger and more diverse sample sizes (including medical students) from across various institutional settings and which applies more rigorous study designs (i.e. pre-test post-test with a control group; second post-test after students have begun their clinical positions).

## Additional file

Additional file 1: Patient safety sample case scenario. Example of a patient safety case scenario that was used during Team-Based Learning activities in the classroom. (PDF 153 kb)

## Abbreviations

GRAT: Group Readiness Assurance Test
IPE: Interprofessional Education
iPEPS: Interprofessional Education in Patient Safety
IRAT: Individual Readiness Assurance Test
TBL: Team-Based Learning
TBL-SAI: Team-Based Learning Student Assessment Instrument
UWE-IP-D: University of the West of England’s Interprofessional Questionnaire – German version
WHO: World Health Organization
