# Supplementary material for: Applying interprofessional Team-Based Learning in patient safety: a pilot evaluation study
Source: BMC Med Educ. 2018 Mar 27;18:48. doi: 10.1186/s12909-018-1164-8 (PMC5870187; doi:10.1186/s12909-018-1164-8)
Supplement: Supplementary file 1 — Patient safety sample case scenario. Example of a patient safety case scenario that was used during Team-Based Learning activities in the classroom. (PDF 153 kb) [file 12909_2018_1164_MOESM1_ESM.pdf]

MEED-D-16-00819, Revised Manuscript

Applying Team-Based Learning for interprofessional education in patient safety: A pilot evaluation study

Lukas Lochner, Sandra Girardi, Alessandra Pavcovich, Horand Meier, Franco Mantovan, Dietmar Ausserhofer

Additional file: Patient safety sample case scenario

An occupational therapist is walking to the bathroom with a 74-year-old patient who has hemiparesis on the left side. The patient is scheduled to have a treatment session in the bathroom to regain his autonomy. On this particular day, he also has a slight cold, with some difficulty breathing. The bathroom is at the end of the corridor. After the treatment session, on the way back to his room, the patient is exhausted and so the therapist allows him to sit in a chair in the corridor. He tells the patient to remain seated while he leaves to get a wheelchair. The patient, however, decides to get up and walk back to his room on his own. On the way, he hits his head on an overhead sign, scraping his temple. A nurse passing by at that moment helps the patient, making him sit down again. The patient is now agitated and has some difficulty breathing. The nurse tries to calm him down and examines his head. She notices a mild bruise and small cut. In the meantime, the occupational therapist returns. Together, they help the patient get into the wheelchair and set him back up in his room. They call the doctor right away, who confirms the diagnosis: mild haematoma and a small cut on the right temporal side of the head. The patient’s wound is dressed.
